# Petersen’s Hernia With a Twist

**DOI:** 10.7759/cureus.78370

**Published:** 2025-02-02

**Authors:** Jake Clements, Barry W Clements, Joshua M Clements

**Affiliations:** 1 Surgery, Belfast Health and Social Care Trust, Belfast, GBR

**Keywords:** gastric bypass surgery, laparoscopy, late complication, petersen's hernia, superior mesenteric vein occlusion

## Abstract

Petersen’s hernia is a well-recognized idiosyncratic complication of gastric bypass surgery for morbid obesity. Mesenteric vein thrombosis is a much rarer clinical entity and is generally associated with an underlying thrombophilia. We present a patient who developed a Petersen’s space hernia with concomitant superior mesenteric vein thrombosis following a laparoscopic Roux-en-Y gastric bypass procedure. We discuss the pathophysiology of both conditions and a potential causal relationship.

## Introduction

Petersen’s hernia is the most common form of internal hernia occurring after laparoscopic Roux-en-Y gastric bypass (LRYGB) for morbid obesity, especially when an antecolic approach is employed [1]. Mesenteric vein thrombosis is a much rarer clinical entity. To date, associations have been made with underlying thrombophilia, such as protein C and S deficiency, antithrombin III deficiency, plus high levels of clotting factor VIII [2]. We present a patient who developed Petersen’s space hernia with concomitant superior mesenteric vein (SMV) thrombosis following LRYGB. We discuss the pathophysiology of both conditions independently and postulate why SMV occlusion is much more likely in the setting of Petersen's hernia. The authors stress the importance of recognizing the risk of concomitant SMV occlusion in the setting of Petersen's hernia, which compounds the risk of serious morbidity, and highlight the importance of making the diagnosis expediently.

## Case presentation

A 28-year-old female with a BMI of 55 kg/m² underwent an uncomplicated LRYGB. Fifteen months later, her BMI had reduced to 28 kg/m², representing a total excess weight loss of 70%. She presented to her district general hospital complaining of a weeklong history of colicky postprandial epigastric pain. She was not using the oral contraceptive pill, and her history was otherwise unremarkable. Vital signs were unremarkable. Abdominal examination revealed mild epigastric tenderness with normal bowel sounds. Despite this, the pain was unremitting, and the patient required regular narcotic analgesia. On admission, baseline blood tests (FBC, U+E, LFT, serum amylase, and clotting screen) were within normal limits (Table 1).

**Table 1:** Blood results on admission eGFR: estimated glomerular filtration rate, CRP: C-reactive protein, Hb: hemoglobin, WBC: white blood cells, APTT: activated partial thromboplastin time, ALP: alkaline phosphatase, ALT: alanine transaminase, GGT: gamma-glutamyl transpeptidase, AST: aspartate transaminase

Test	Value	Normal range
Urea	6.8 mmol/l	2.5-7.5 mmol/l
Creatinine	85 umol/l	80-110 umol/l
Sodium	140 mmol/l	135-145 mmol/l
Potassium	4.2 mmol/l	3-5 mmol/l
eGFR	>60 ml/min	>60 ml/min
CRP	4.3 mg/l	<5mg/l
Hb	127 g/l	120-160 g/l
WBC	8.2 x 109/l	3.6-11 x109/l
Platelets	243 x 109/l	140-400 x 109/l
Prothrombin time	13 sec	9-13 sec
aPTT	35 sec	22-36 sec
Amylase	93 U/dl	28-100 U/dl
Bilirubin	20	<21 umol/l
ALP	109 U	30-130 U/l
ALT	27	<33 U/l
GGT	17	<40 U/l
AST	22	1-45 U/l
Albumin	43	35-50 g/l

**Figure 1:**
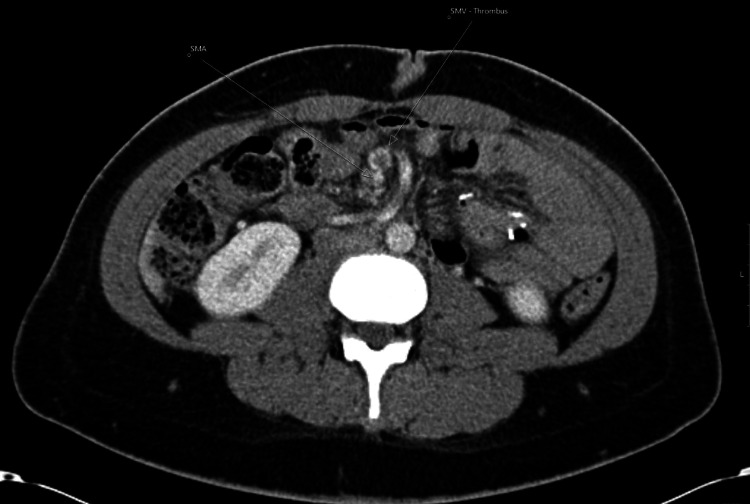
CT scan demonstrating SMV containing thrombus (arrow) and normal SMA (arrow) CT: computed tomography, SMV: superior mesenteric vein, SMA: superior mesenteric artery

A contrast-enhanced spiral CT scan of the abdomen was performed at the primary hospital, and radiological findings showed a thrombus in the SMV with “malrotation of the midgut” (Figure 1). The referring service had no experience with bariatric surgery, so the recognition of the potential association with previous bariatric surgery had not been considered. On these grounds, the patient was commenced on therapeutic enoxaparin and referred to a tertiary center.

**Figure 2:**
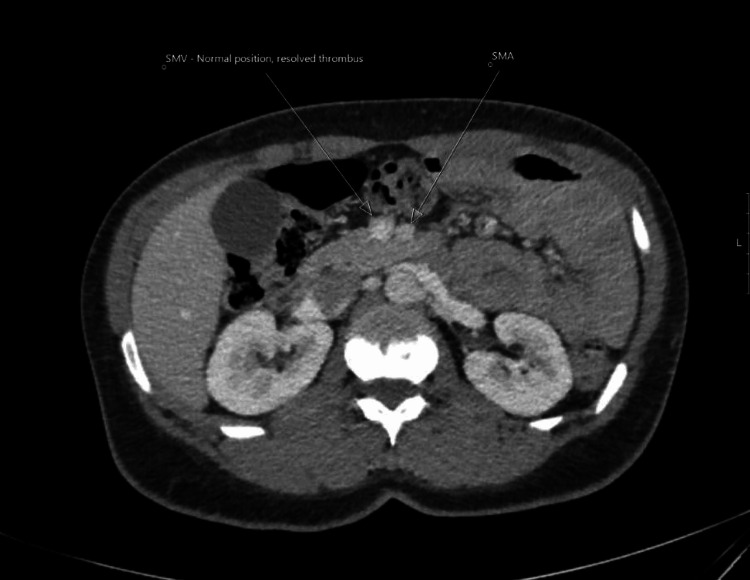
CT scan demonstrating normal SMV showing thrombus resolution (arrow) and SMA (arrow) CT: computed tomography, SMV: superior mesenteric vein, SMA: superior mesenteric artery

The receiving surgeon had bariatric surgical experience and dismissed the diagnosis of "malrotation" in favor of the eponymous Petersen’s hernia with SMV occlusion. Consequently, the immediate laparoscopic assessment was performed, and the diagnosis was confirmed, revealing herniation of the pancreaticobiliary limb from left to right through Petersen’s space (Figure 2). The small bowel appeared dilated; however, there was no overt evidence of engorgement or ischemia. The hernia was reduced, and the defect was closed with non-absorbable suture material.

**Figure 3:**
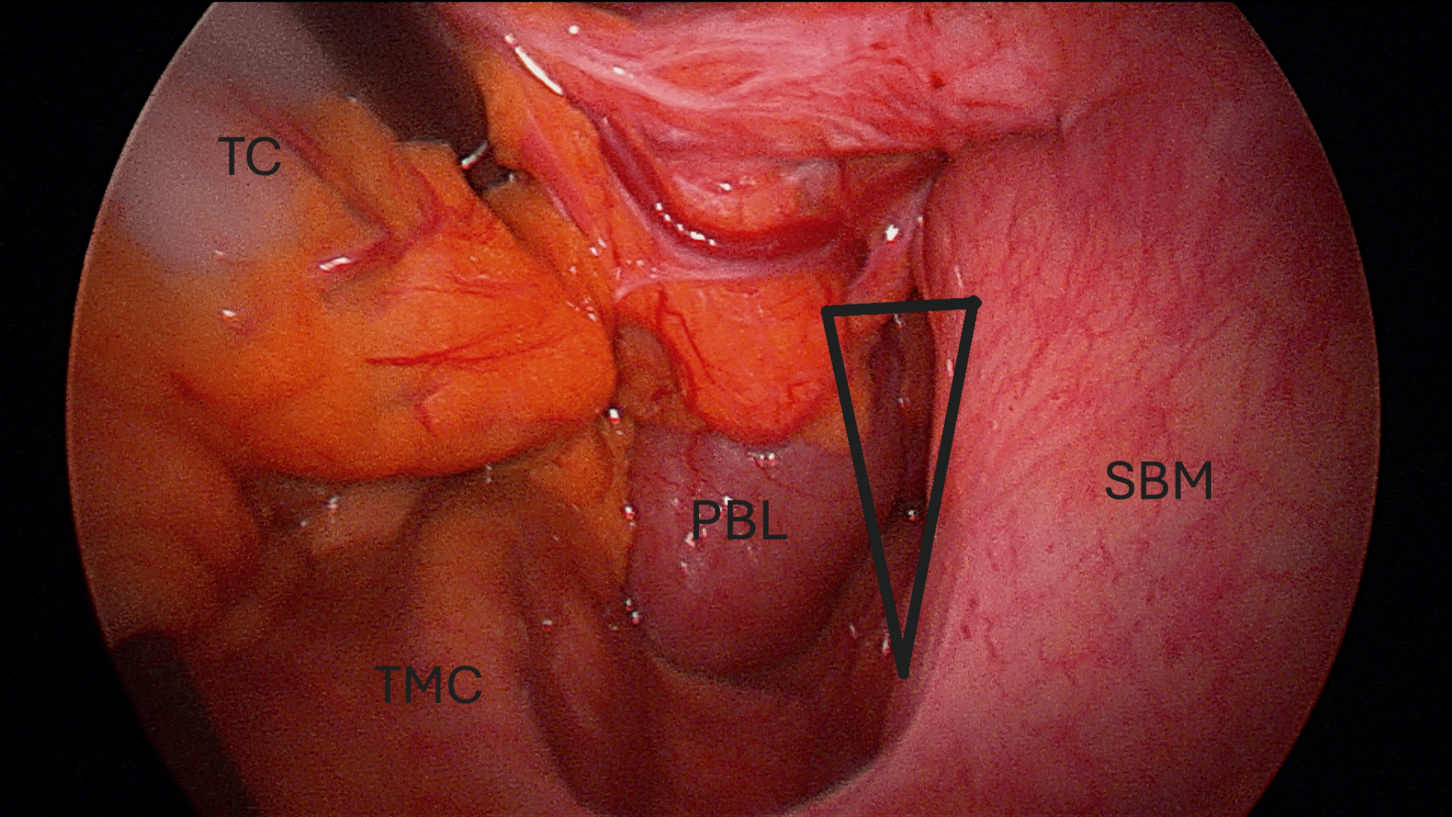
Operative photograph demonstrating boundaries of Petersen's space (Triangle), TC, TMC, SBM, and PBL TC: transverse colon, TMC: transverse mesocolon, SBM: small bowel mesentery, PBL: pancreatobiliary limb

The patient made an uneventful recovery and was discharged on the second postoperative day, having been commenced on warfarin. She has since been reviewed, and a follow-up CT scan has demonstrated complete resolution of the SMV thrombosis (Figure 3). Subsequent coagulation studies have excluded the presence of an underlying thrombophilia.

## Discussion

Morbid obesity is a global problem, with an incidence of 14% in females and 8 % in males [3]. To date, the only long-term effective treatment strategy for combating this problem is surgery [4]. The two principal approaches in bariatric surgery are gastric restriction with or without intestinal bypass. LRYGB is still one of the most commonly practiced bariatric procedures; however, where gastric banding has fallen out of favor, sleeve gastrectomy has emerged as the most commonly practiced procedure [5]. Studies have shown that approximately 25% of patients will develop complications [6], many requiring urgent intervention. The vast majority of these complications are early complications such as an anastomotic leak, bleeding, and consequent sepsis; however, the rare complication we describe usually occurs much later and hence often falls off the clinician's radar. This patient population usually attends their local emergency department, where there is little or no experience of postoperative bariatric surgical complications.

An internal hernia is the protrusion of abdominal viscera, most commonly small bowel loops, through a peritoneal or mesenteric aperture into a compartment within the abdominal and pelvic cavity [7]. Hernial orifices can be congenital, including both normal foramina or recesses and unusual apertures resulting from anomalies of peritoneal attachment and internal rotation, or acquired if caused by inflammation, trauma, and previous surgery, like gastric bypass for bariatric treatment and liver transplantation. The overall incidence of internal hernias has been increasing in recent years [8]. Although relatively uncommon, they represent a potentially life-threatening condition with bowel entrapment and, if left untreated, progression to strangulation and ischemia. According to various investigators, internal hernias cause up to 5.8% of all small bowel obstructions [9], with a mortality rate in excess of 50% [10]. Three types of Roux-en-Y-related hernias have been described: "transmesocolic," the most common, in which the bowel loops herniate through the surgical defect in the transverse mesocolon with possible mass effect on the stomach and displacement of the transverse colon anterior-inferiorly [11]; "jejunostomy mesenteric," in which bowel prolapses through a defect in the small-bowel mesentery of the jejunojejunostomy site; and finally, the "Petersen type" [12], in which bowel loops protrude behind the Roux loop before the small bowel eventually passes into a space called the Petersen defect, located between the jejunal mesentery of the Roux limb and transverse mesocolon. A deformed and displaced Roux limb, biliopancreatic limb, and transverse colon may serve as landmarks of these hernias.

Petersen’s hernia is typically an intermediate or late complication with the patient losing weight and consequent space enlargement. The diagnosis is elusive as the clinical presentation is often insidious and nonspecific. The alimentary limb and common channel are usually the intestinal segments involved in Petersen's hernia, although the pancreaticobiliary limb can also be culpable, as in the case presented. The symptoms of closed-loop obstruction can result in a vague symptom complex with diffuse abdominal pain and nausea. Radiological investigations are often inconclusive, and the only way to diagnose this condition confidently is by adopting a high index of clinical suspicion and an expedient laparoscopy by an experienced surgeon. This is especially important when the offending hernia can alter or compress the mesenteric root, resulting in flow disturbance in the SMV, which, at worst, can result in venous infarction of the whole small intestine. Closure of the mesenteric defects with sutures or clips has evolved as a positive intervention and reduces the risk of Petersen's hernia [13]. The downside of the sutured approach is that it can add length to the original surgery. It has an increased risk of early postoperative complications, including torsion of the small bowel near the jejunojejunostomy, resulting in partial or complete obstruction of the alimentary or the biliopancreatic limb. Closure of the mesenteric defects with clips has been reported to be quicker and to have a lower risk of postoperative complications [14].

CT is the gold standard investigation in emergency abdominal conditions, particularly where small bowel pathology is suspected. It has a high specificity and sensitivity in identifying the site, level, obstruction cause, and ischemic changes in the involved bowel. With the possibility of using high-quality three-dimensional reformation techniques, CT provides important advantages in evaluating small bowel and surrounding structures, increasing diagnostic confidence in the transition zone localization [15]. Small bowel obstruction of an internal hernia is usually a closed-loop obstruction, in which a segment of the bowel is occluded at two adjacent points along its course. Direct signs of a closed loop at CT are a U- or C-shaped, fluid-filled, distended intestinal loop or a radial array of distended loops with stretched and thickened mesenteric vessels converging to a central point. In this setting, a cluster of dilated loops, or a "sac-like appearance" of crowded small bowel loops owing to encapsulation within the hernia sac at an abnormal anatomic location, highly suggests an internal hernia [16]. The "mesenteric swirl" CT findings with small bowel obstruction are the classical CT signs for Petersen's hernia. In addition, SMV beaking (a decreased caliber of the SMV with a beaked appearance) and the "criss-cross" appearance of the second-order mesenteric vessels with reversal of the SMV and SMA anatomic relationship have been described more recently [17].

Where morbid obesity is associated with an increased risk of peripheral thromboembolism, visceral thromboembolism is a rare clinical entity that is not seen any more commonly in obese patients than those with a normal BMI [18]. SMV and portal vein occlusion are often associated with thrombophilic conditions such as protein C, protein S, and antithrombin III deficiency. We postulate that with the "twisting" of the mesenteric root that may result from the offending intestinal segment, the blood flow through the SMV can be adversely affected, resulting in secondary thrombosis. The secondary development of this thrombotic complication associated with Petersen's hernia compounds the time pressure on clinicians to consider this illusive diagnosis so treatment can be affected expediently.

## Conclusions

Intestinal ischemia is not uncommon in internal herniation, where a closed-loop obstruction is not diagnosed expediently. This is normally the result of compression of the vascular inflow or impedance of venous drainage and is usually segmental. Intestinal ischemia due to SMV occlusion in the setting of Petersen's hernia is a very rare complication, with only sporadic reports in the literature. This is felt to have a different pathophysiology where the root of the superior mesenteric vascular bundle is distorted due to the space-occupying effect of the hernia. With impaired flow in the SMV, thrombosis occurs. This has the potential for catastrophic, irreversible intestinal ischemia and death. With the exponential increase in bariatric surgery worldwide, it is vitally important that all clinicians are familiar with this idiosyncratic complication associated with Petersen's hernia if serious sequelae are to be avoided.
